# Oil price shocks, economic policy uncertainty and industrial economic growth in China

**DOI:** 10.1371/journal.pone.0215397

**Published:** 2019-05-10

**Authors:** Jingyu Chen, Faqi Jin, Guangda Ouyang, Jian Ouyang, Fenghua Wen

**Affiliations:** 1 School of Business, Central South University, Changsha, China; 2 Washington University in St. Louis, One Brookings Drive, St. Louis, MO, United States of America; 3 Changsha Research Institute of Mining and Metallurgy Co., Ltd, Changsha, China; 4 Supply Chain and Logistics Optimization Research Centre, Faculty of Engineering, University of Windsor, Windsor, ON, Canada; 5 Centre for Computational Finance and Economic Agents, University of Essex, Colchester, United Kingdom; The Bucharest University of Economic Studies, ROMANIA

## Abstract

This paper combines a Granger causality test and a VAR model to investigate the relationships among oil price shocks, global economic policy uncertainty (GEPU), and China’s industrial economic growth. Based on monthly data from 2000 to 2017, we reveal that GEPU and world oil prices jointly Granger cause China's industrial economic growth; world oil prices have a positive effect on China's industrial economic growth, while GEPU has a negative effect. Further analyses investigate the asymmetry effect of oil prices and find that the negative component shows a more significant impact on China's industrial economic growth. The results are robust to different oil price and EPU proxies.

## 1. Introduction

Crude oil, the black blood of industry, plays an important role in economic growth [1–4] and inspires the great interest of researchers [5–9]. Given the importance of crude oil, there is also attention paid to what would happen to the economy when its price changes. The classical theory about the relationship between oil and economic growth is based on supply-side theory. As an indispensable raw material in production, oil price determines the oil’s cost. Producers would adjust their production plan to achieve the maximum profit, which would affect output and in turn affect the macroeconomy (see, e.g., [10–13]).

**Fig 1:**
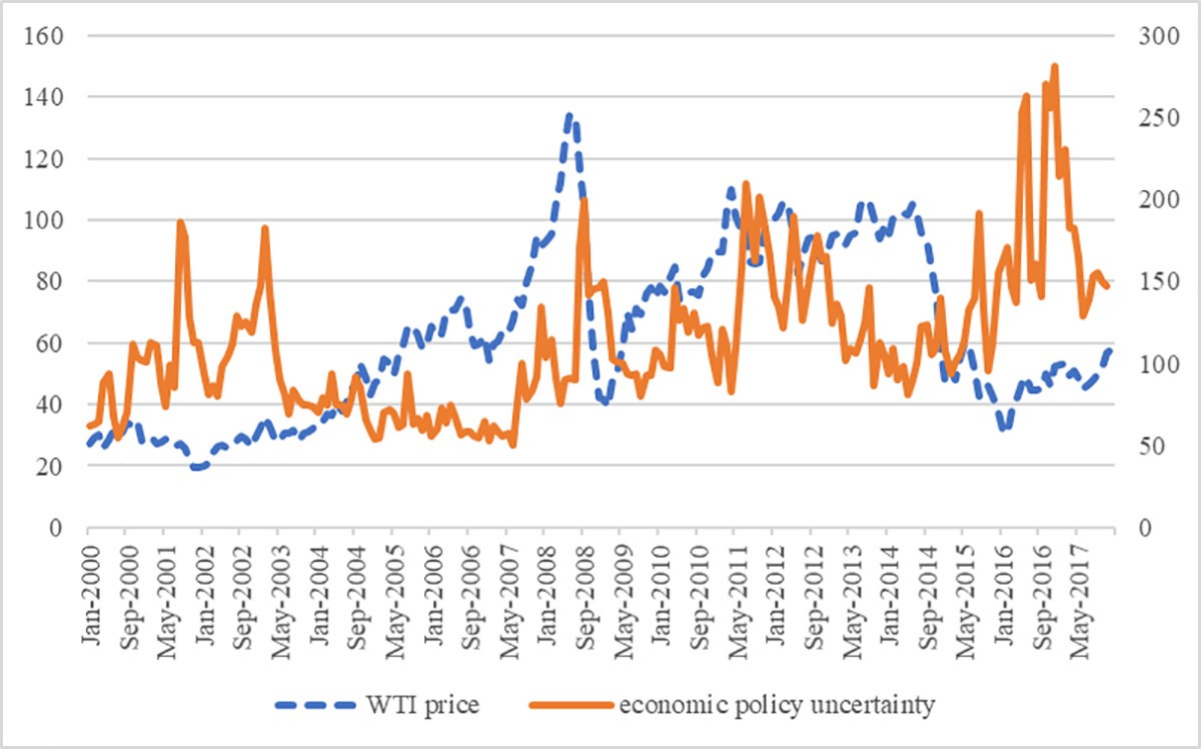
The real oil price and economic policy uncertainty: 1998M1-2016M12.

This study builds on the work of Kang and Ratti [14]. In their pioneering work, Kang and Ratti examine the causal effects of oil price shocks and economic police uncertainty, and find that oil price shocks and economic policy uncertainty are interrelated and jointly influence stock market. Derived from Kang and Ratti [14], Antonakakis et al. [15] examine the spillover effect of oil price shocks and economic police uncertainty. They find that economic policy uncertainty and oil price shocks response to each other negatively. In addition, they become the dominant transmitters since 1997 in the net term. As shown in Fig 1, the real oil price climb to the peak in an unprecedented rate since 2007 and suffers from a large decline in 2008. At the same time, economic policy uncertainty sharply decreases and rebound after 2008. In addition, economic policy uncertainty shows the opposite trend to oil price most of the time. These phenomena imply that oil price shocks may be related to economic policy uncertainty, and it is along with the work of Kang and Ratti[14].

Before Baker et al. [16], there was no standard method to measure these exogenous events related to uncertainty. To solve this problem, the authors construct an index of economic policy uncertainty (EPU) formed by three components—a News Index, a Tax Expiration Index, and an Economic Forecaster Disagreement Index. Based on the EPU index, Aloui et al. [17] find that changes in the EPU can distinguish oil price shocks from supply shocks, demand shocks and specific demand shocks, thus directly affecting oil price. That is, a unit increase (decrease) in uncertainty will negatively (positively) affect economic activity and, hence, decrease (increase) the demand for and supply of crude oil. This propagation process can result in fluctuations in oil prices. Antonakakis et al. [18] investigate the relationship between volatility and the EPU index and find that the total demand shock of oil prices has a negative dynamic relationship with US economic recessions and the stock market. Meanwhile, attention also has been paid to the relationships between economic policy uncertainty and macroeconomy. Economic policy uncertainty has a great influence on macroeconomies at the macro and micro levels. On the one hand, policy makers would pay more attention to economic growth because of its critical position [19–21]. On the other hand, changes in the economy and policy mean risks, which would affect the decision firms make [22–25]. At the same time, the supply and demand relationship will change when facing risks, as seen in interest rate, inflation and expected risk premiums [26–29]. A growing body of research has explored the influence of economic policy uncertainty on macroeconomies [30–32]. Considering the relationships between oil price and economic policy uncertainty, the absence of either one will lead to endogenous problems [33].

At present, there is little evidence available on the effect of oil price and economic policy uncertainty on economic growth. Especially, as one of the emerging economies in the world, China shows tremendous demand for crude oil. By 2015, the proportion of crude oil consumption in China's energy structure had reached 18.1%. The dependence on imported oil is also increasing year by year: from 30.6% in 2002, it increased to 56.5% in 2010 and then reached 67.4% in 2017. China’s Petroleum Industry Development Report (2018) reported that, in 2017, China surpassed the United States as the world's largest crude oil importer for the first time and has remained that way since then. Du et al. [34] find that the world oil price affects China’s economic growth and inflation significantly while the latter is fails to affect world oil price. Gong and Lin [35] find that oil shocks from different sources have different impacts on China’s output and inflation, and these effects are time-varying. Wei [36] find that oil price can significantly affect the real export and trade terms in China, and economic policy uncertainty shocks play the most important role in accounting for the variance of China’s trade. These phenomenon and studies show that the economies of industrialized countries will continue to rely on oil, and the changes in world crude oil price will affect China's industry more deeply [37–39]. Meanwhile, increasing global trade and political communications make it impossible to be an isolated economy. In this sense, global economic policy uncertainty (GEPU) shows its irreplaceable meaning for China. Therefore, only by integrating the effect of oil price shocks and economic policy uncertainty into one system can China’s industry cope with oil price shocks more calmly.

Additionally, since Mork [40] finds that an increase in the price of oil has a greater influence on a country’s macroeconomy than a decrease, the asymmetric effects of oil prices have grabbed the attention of researchers [41–46]. Mork et al. [47] conclude that an increase in the oil price has a negative effect on GDP, whereas a decrease in the oil price has not been found to have a positive impact on output or an impact to the same degree. Hu et al. [48] analyze the short-run and long-run asymmetric impact of oil price shocks on China’s stock market and find that the demand-side shocks have a significant impact in both the short and long run and that only the aggregate demand shock has an asymmetric effect. Therefore, a study about the asymmetric effect of crude oil with a framework considering the economic policy uncertainty index also attracts our attention.

As far as we concerned, no study has established the relationships between oil price shocks, economic policy uncertainty, and economic growth in China’s industry. Therefore, our contribution is to supplement this research. To be more specific, we fill the literature gap from the following aspects: (a) we examine the joint effects of oil price shocks and economic policy uncertainty on economic growth in China. The theoretical literature recognizes that oil price changes and economic policy uncertainty may have a joint effect of on macroeconomy, but few empirical studies have analyzed this issue satisfactorily; (b) to study the asymmetric effects of oil price shocks, we decompose the oil price shocks into positive and negative parts. Few studies prove that the effect from oil price decrease increase economic growth, but oil increase could slow down economic development. Hence, there might be asymmetric effects of oil price shocks on the economic growth. Based on the above discussions, we investigate the influence of crude oil prices and economic policy uncertainty on China’s industrial economic growth. By constructing a VAR model, we first examine the Granger causality and then apply impulse response functions to specify the impact. Furthermore, under the framework considering EPU, we decompose the oil price shocks into positive and negative parts and examine their contribution. To verify the results, we consider two robust tests and make conclusions.

The remainder of this paper is organized as follows. Section 2 explains the empirical methodology; Section 3 provides a description of the data and preliminary analysis; Section 4 presents the empirical results; and Section 5 considers the structural stability issue. We conclude in the last section.

## 2. Empirical methodology

The VAR model is adapted to examine the effect of oil price shocks and global economic policy uncertainty on China’s industrial economic growth. Oil price changes can influence the demand for industrial output, and economic policy uncertainty can change the views of investors. Moreover, oil price shocks and economic policy uncertainty co-affect economic growth by influencing the expected risks to the environment and the expected relationship between supply and demand.

Since Sims [49] originated the VAR approach, it has been the standard tool for examining the oil price shock mechanism. A VAR model is an economic model used to capture the linear interdependencies among multiple time series and allows for each variable to be treated as endogenous, thus avoiding restrictions. VAR models have been used in determining and analyzing the relationship between oil prices and economic indicators. The first important feature of the VAR model is its flexibility and simplicity in generalization.

In this paper, consider the following VAR model of order *p*:

$$Y_{t}=C+\Theta_{1}Y_{t-1}+\cdots +\Theta_{p}Y_{t-p}+\varepsilon_{t},\;t=1,2,\cdots ,\;T$$ (1)

where Y_t_ = (R_t_, GEPU_t_, IAV_t_) is a 3×1 vector of endogenous variables, while *Y*_*t*−*p*_ is the corresponding lag terms of order p, which are determined by the SC information criterion. *Θ*_*p*_ is the 3×3 matrix of autoregressive coefficients of vector *Y*_*t*−*p*_ for i = 1, 2, …, p. c = (c_1_, c_2_,…c_*n*_)′ is the 3×1 intercept vector of the VAR model. ε_*t*_ = (ε_1*t*_, ε_2*t*_,…ε_*nt*_)′ is the 3×1 vector of the white noise process.

Since the technique for estimating the standard error is inaccurate, the estimated coefficients of the VAR model usually seem to lack significance. In this case, we apply impulse response functions to display the dynamic effects of endogenous shocks. Usually, a one-standard-deviation shock will be given, and then, we will test the effect on the endogenous variables and their future values.

The fundamental rationales are as follows.

Define L as the lag operator (LY_t_ = Y_t-1_). We can derive from (1) as follows:

$$(I_{k}-\Theta_{1}L-\Theta_{P}L^{p})Y_{t}=\varepsilon_{t}$$ (2)

$$Y_{t}=(I_{k}-\Theta_{1}L-\Theta_{P}L^{p}){}^{-1}\varepsilon_{t}=\left(I_{k}+C_{1}L+C_{1}L^{2}+\cdots \right)\varepsilon_{t}$$ (3)

For example, IAV (the *industrial added value*, one of the key variables in this study. Section *3*.*1 data description*) can be written as:

$$IAV_{t}=Y_{3t}=\sum_{j=1}^{3}(c_{3j}^{\left(0\right)}\varepsilon_{jt}+c_{3j}^{\left(1\right)}\varepsilon_{jt-1}+\ldots ),\;t=1,2,\ldots ,T$$ (4)

$c_{3j}^{\left(q\right)}$ is the element of *C*_*q*_ on the third row and jth line. This function shows the response of IAV to a shock of another variable, holding all other variables constant. At the same time, we should construct an orthogonal matrix to transform shocks from correlated to uncorrelated in the same period.

Next, we use variance decomposition to estimate the importance of different structural shocks.

Taking IAV as an example, the variances are as follows:

$$E\left[(c_{3j}^{\left(0\right)}\varepsilon_{jt}+c_{3j}^{\left(1\right)}\varepsilon_{jt-1}+c_{3j}^{\left(2\right)}\varepsilon_{jt-2}\ldots {)}^{2}\right]=\sum_{q=0}^{\infty }(c_{3j}^{\left(q\right)}{)}^{2}\sigma_{jj},\;j=1,2,3$$ (5)

If there are no correlations between variables, the contribution of various disturbances to variance (RVC) is denoted as the following proportion:

$$\mathrm{RVC}=\frac{\sum_{q=0}^{\infty }(c_{3j}^{\left(q\right)}{)}^{2}\sigma_{jj}}{\sum_{j=1}^{3}\sum_{q=0}^{\infty }(c_{3j}^{\left(q\right)}{)}^{2}\sigma_{jj}},\;j=1,2,3$$ (6)

## 3. Data description and preliminary analysis

### 3.1 Data description

Industry, as the main material production department in China, occupies the largest share of GDP. Industrial added value (IAV) is an important indicator to measure a country's industrial development level. At the same time, it is also a core indicator of the national economic accounting system. The National Bureau of Statistics of China only publishes IAV data monthly, and the year-to-year data on January after 2006 are unavailable. We use interpolation to fill in the data gaps. The data are derived from the website of the CMSAR China financial database. CMSAR draws on the experience of various well-known foreign databases and combines China’s national conditions. It is the largest and most accurate financial and economic database in China. For the oil price, we choose the West Texas Intermediate Spot Price (WTI), which is derived from the U.S. Energy Information Agency database (EIA), as the proxy for the world oil price. Furthermore, we use return (R), which is defined as follows, for later study; thus, any effect on the exchange rate will be reduced:

$$R=ln(P_{t})-ln(P_{t-1})$$ (7)

Additionally, to investigate asymmetric effects, we divide oil price shocks into positive and negative components according to the work by Schorderet [50]. The nonlinear transformations are presented as follows:

$${os}^{+}=\max\left(0,R\right)$$ (8)

$${os}^{-}=min(0,R)$$ (9)

The index of economic and policy uncertainty (EPU) includes monthly data and is derived from the website www.economic policy uncertainty.com. These variables consist of 216 observations from 2000M1 to 2017M12, and the detailed descriptions of all the variables and data sources are presented in Table 1.

**Table 1:** Variable definitions.

Variables	Definitions of variables
R	First log difference of monthly stock price (WTI) ^a^
GEPU	Log transformation of global economic policy uncertainty ^b^
IAV	Log transformation of China’s industrial added value ^c^
os^+^ = max (0,R)	Positive rate of change in the oil price, ${os}^{+}=\left\{ \begin{array}{c} R,\;R>0\\ 0,R<0 \end{array}\right.$
os^−^ = min (0,R)	Negative rate of change in the oil price, ${os}^{-}=\left\{ \begin{array}{c} 0,\;R>0\\ R,R<0 \end{array}\right.$

Data sources:

^a^ https://www.eia.gov/dnav/pet/hist/LeafHandler.ashx?n=PET&s=RWTC&f=M

^b^ http://www.policyuncertainty.com/global_monthly.html

^c^ http://www.gtarsc.com/SingleTable/DataBaseInfo?nodeid=4120

### 3.2 Preliminary analysis

A unit root test is always the initial part to be completed to examine the properties of variables. Table 2 reports the descriptive statistics of our data. Table 3 reports the augmented Dickey-Fuller (ADF) test and Phillips-Perron (PP) test. The null hypothesis of the ADF test and PP test is that the series has a unit root. The results of the unit root test show that almost all variables do not have a unit root at a 1% level of significance (IAV by ADF test at the 5% level). Thus, we accept the assumption that in log levels, the variables are I (0) processes. Therefore, we can choose the VAR model to continue on with the following analysis.

**Table 2:** Statistical description.

Variables	Mean	Median	(Max, min)	Std. Dev.	Skewness	Kurtosis
R	0.0036	0.0162	(0.2602, -0.3948)	0.0932	-0.5711	4.1489
GEPU	4.6565	4.6431	(5.7289,3.9438)	0.3875	0.2916	2.5479
IAV	2.4151	2.4973	(3.1441,0.8329)	0.4067	-0.6834	3.2082
os^+^	0.0386	0.0162	(0.2602,0)	0.0498	1.4866	5.3375
os^−^	-0.0350	0	(0,-0.3948)	-0.0590	-2.3628	10.3117

Note: All variables are expressed as natural log, expect for R.

**Table 3:** Unit root test.

Variables	ADF			PP		
	5% Level	1% Level	t-Statistic	5% Level	1% Level	t-Statistic
R	-3.4310	-4.0015	-12.4040 ^a^	-3.4310	-4.0015	-12.4426^a^
GEPU	-3.4308	-4.0013	-4.9702 ^a^	-3.4308	-4.0013	-4.7122 ^a^
IAV	-3.4321	-4.0039	-3.6650 ^b^	-3.4308	-4.0013	-8.4244 ^a^
os^+^	-3.4309	-4.0015	14.6081 ^a^	-3.4309	-4.0015	14.6462 ^a^
os^−^	-3.4309	-4.0015	-10.9300 ^a^	-3.4309	-4.0015	-10.9163 ^a^

Note: The null hypothesis of the ADF test/PP test is that the series has a unit root.

^a^ Significance at 1%.

^b^ Significance at 5%.

## 4. Empirical results

### 4.1 Granger causality test

To investigate the causal relationships of the variables, we perform Granger causality tests. In a multivariate framework, the first variable is said to cause the second variable in the Granger sense if the forecast for the second variable improves when lagged variables for the first variable are taken into consideration [51]. According to the SC information criterion, the lag was determined as 2 orders, and so do the later empirical tests.

Table 4 displays the Granger causality test for the variables. From it, we can know that the GEPU Granger causes oil prices at the 5% level of significance, while oil prices do not Granger cause GEPU. The relationship between oil prices and IAV shows the same, that is, that oil price shocks Granger cause IAV at the 1% level of significance, but not vice versa. However, IAV and GEPU show bidirectional causality at the 5% and 1% level of significance, respectively. It is worth noting that all the joint equation tests show that there is Granger causality between variables with at least 10% significance. Above all, we conclude that the oil price is Granger caused by GEPU, while GEPU is Granger caused by IAV, and the joint test of any two is the third party's Granger relationship. In particular, oil price shocks and GEPU have a significant Ganger causing effect on IAV, providing the motivation for the use of the VAR model.

**Table 4:** Granger causality test.

Dependent variable	R	GEPU	IVA	ALL
R	…	7.766 ^b^	1.395	9.760 ^b^
GEPU	0.689	…	7.284 ^b^	8.311 ^c^
IVA	12.503 ^a^	9.379 ^a^	…	25.144 ^a^

Note: The null hypothesis that the variables in a row are not significant in explaining the variables in the column is tested. “ALL” denotes the joint test of the variables.

^a^ Significance at 1%.

^b^ Significance at 5%.

^c^ Significance at 10%.

The above results are consistent with our intuition and expectation. Firstly, uncertainty about economic policy decisions negatively affect firm’s decisions about investment and output plan. As a result, it further causes a downward pressure on oil prices, which is the most important raw material in the production. However, the results show that oil prices fail to granger cause economic policy uncertainty. It might because that crude oil price occupies a little place in the economic policy uncertainty index while the latter contains lots of information about the three components (a News Index, a Tax Expiration Index, and an Economic Forecaster Disagreement Index.). Thus, it is not significant that world oil price granger causes the economic policy uncertainty. Secondly, during the past decades, China’s dependence on outsider oil has increased dramatically (reached almost 50% in 2007), and meanwhile, domestic oil price has been more and more related to the world oil price because of the reforms of oil pricing mechanism, it is not hard to find that the world oil price has been influencing China’s economic growth significantly. On the other hand, the share of China’s oil consumption is still relatively small (about 13% in 2016) despite the absolute volume of consumption is large. Therefore, it is not surprising that China still remains little impact on the world oil markets. Consequently, China has not yet got the power of oil pricing in the world oil market though it emerges to be an important player. Thirdly, after decades of development, Chinese industrial added value reached $4.147 billion in 2017, surpass United States, ranking first in the word. From 2010 to 2017, the contribution rate of the Chinese economy to global economic growth has remained above 30%. No wonder, the Chinese industrial economic growth has been influencing global economic policy uncertainty remarkably, and conversely, the global economic policy uncertainty affects China’s macroeconomy obviously.

### 4.2 Impulse response analysis

**Fig 2:**
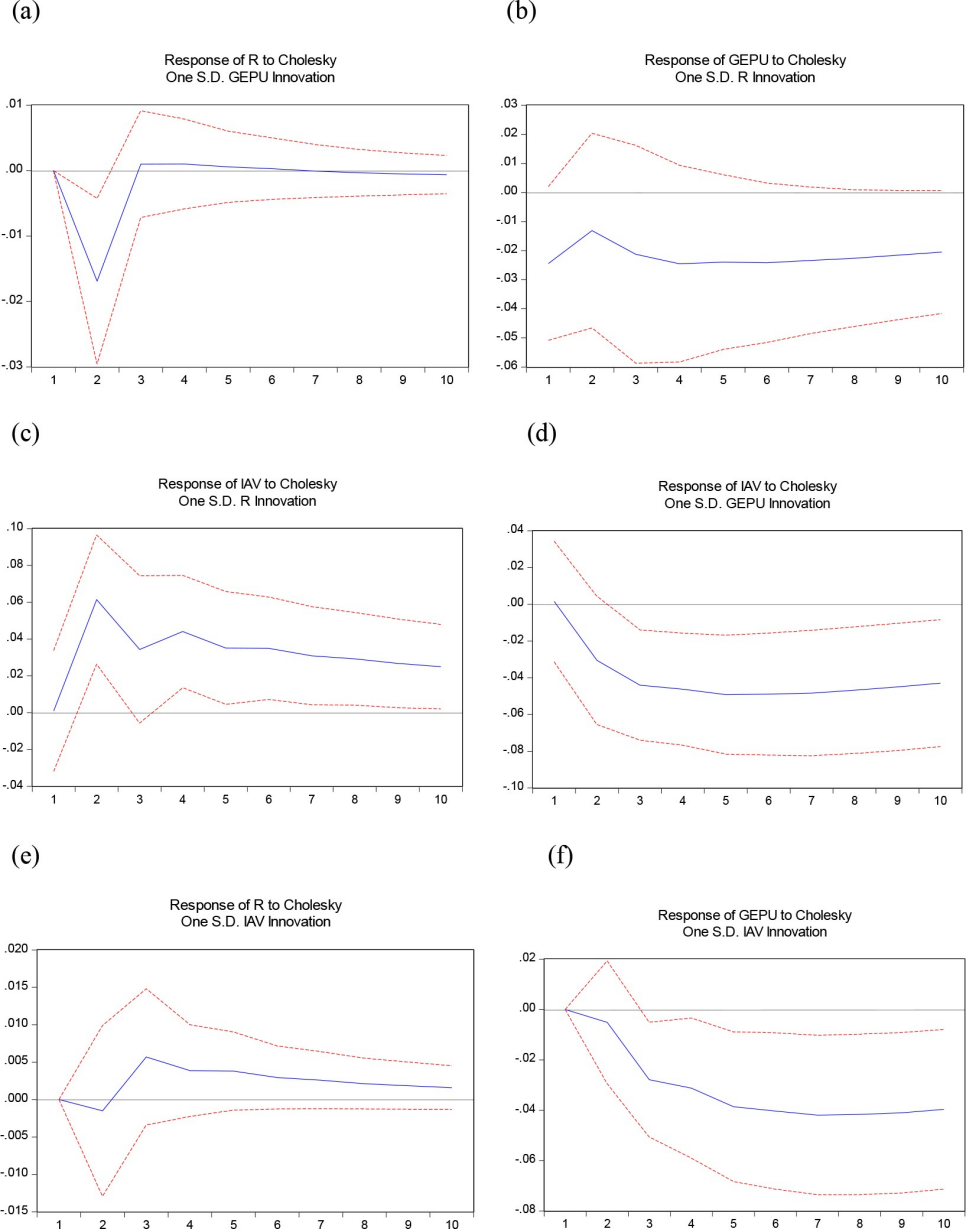
Impulse response shock analysis. Note: In VAR (R, GEPU, IAV), GEPU and IAV are the global economic policy uncertainty and China’s industrial added value in first log difference, and R is real stock return of world real oil price (WTI). The horizontal axis is the period. The vertical axis is the explanation level of the dependent variables to independent variables. In the model, we fix the periods at 10 months.

In the previous section, the Granger causality test shows if there exist any relationships among the variables. Furthermore, this section uses the impulse response functions (IRFs) to test the effect of a one-standard-deviation shock on the endogenous variables and their future values, which clarifies the direction and the extent of the impact. Fig 2A displays the estimated impulse response of oil price to GEPU, and it shows a negative response and reaches the maximum at the second period. The negative response gradually weakens and is followed by a weakly positive response, which reaches the top at the third period, before finally converging steadily.

Fig 2A shows that unanticipated innovations in economic policy uncertainty will decrease international oil prices in the short term This result is consistent with our cognition. When markets become volatile economy, decision makers will choose a more robust investment approach. Once demand tightens, oil price will fall down thanks to the market mechanism.

As Fig 2B depicts, the response of GEPU to an oil price shock decreases in the second period and returns to the previous level in the next period, eventually weakening at a slower rate of decline, which implies that a surprise increase of oil price shock can greatly suppress global economic policy uncertainty. It is counter-intuitive that economic policy uncertainty responds negatively to positive changes in oil prices. According to Antonakakis et al. [15], this result is expected because the supply-side shocks are no longer important for macroeconomic developments. Rises in aggregate demand are regarded as positive information that reflecting prosperity and thus lowering economic policy uncertainty while oil price is pushed upwards.

Fig 2C and 2D depict the response of IVA to international oil price shocks and GEPU, respectively. Fig 2C shows that the IAV responds positively immediately, while oil price has a surprise increase. This reaction will last for five periods and slowly weaken to a stable state. It can be seen that the impact of oil prices has a positive effect on economic growth and will promote China's economic growth. Unlike most studies in the exciting literature, as an oil importing country, a surprise increase of world oil price should have improved the production cost of industry in China, but this does not seem to happen. The reasons for the positive response may be concluded as follows, (1) Our finding is strongly consistent with those of Du et al., Cunado et al. and Wei and Guo [34,52,53]. Reviewing these related papers, it is not difficult to find that the selection of key macroeconomic indicators undoubtedly affects the conclusions. (2) As Cross and Bao [54] described in their research, “while oil supply and specific oil demand shocks tend to have negative movements in China’s GDP growth, oil demand shock generally produces positive effects”. It means that the fast development of China in the 21st Century has created an enormous demand for oil. In this way, the oil price shocks on China’s economic growth present positively. (3) As Chen and Lv [55] mentioned in their paper, thanks to the Chinese special refined oil pricing mechanism, the negative impact of oil prices on economic growth will slow down. Meanwhile, Kilian and Park [56] declared “business cycle theory”–as the most important commodity, crude oil is an indicator of the state of the macroeconomy. Especially, the innovation of global business since the start of the 21st Century takes stimulus in the crude oil market and economic growth as continuous.

As the response of IAV to GEPU painted in Fig 2D describes, when economic policy uncertainty increases, economic growth slows, reaches the minimum -0.5 at the fifth period, and finally converges to a steady state. This result indicates that unanticipated innovations to economic policy uncertainty will decrease China’s industrial economic growth rate. The reason is that economic and political stability is an important prerequisite for the steady development of the economy. Once the risk increases, it will have a negative impact on economic development. To reduce the losses caused by uncertainty, industrial producers will adopt conservative production plans to reduce output.

Finally, to further explore the impact of China's industrial economic growth on international oil prices and economic policy uncertainty, this paper analyzes the response of international oil prices and economic policy uncertainty to China's industrial economic growth. Fig 2E and 2F depict the impact of IVA on international oil price shocks and GEPU, respectively. It can be seen from Fig 2E that in the initial stage, when the industrial economic growth is positively impacted by one unit in the current period, the international oil price will have a negative response, but after the second period, the negative response gradually weakens and turns positive. The response reached its maximum value in the third period and, finally, gradually converged and stabilized. This shows that as a factor of production, the economic benefits will increase demand and thus raise oil prices. The impulse response in Fig 2F shows that when economic growth is affected by a unit of positive impact, the international economic policy uncertainty response is immediately negative. The speed of the response increases sharply in the second period and then slows down, gradually increasing to the maximum value in the eighth period. Finally, it converges gradually. It is depicted that, after a favorable increase in economic growth, global economic policy uncertainty decreases significantly. This may because that a prosperous economy means a more stable economic and political environment.

In retrospect, we find that there is a mutually inhibiting relationship between international oil prices and EPU. The response from oil prices is expected, given that increased EPU may lead to lower productivity and thus lower demand for oil. These results are in line with those of Kang and Ratti [14]. The increase in economic policy uncertainty risks will inhibit the growth of China's industrial economy, while the rise of international oil prices plays a catalytic role. The output tends to be conservative when facing risks, and the positive response to oil price shocks is consistent with Du et al., Cunado et al. and Wei and Guo [34,52,53]. Additionally, oil demand shocks, which produce positive effects on industrial economic growth, may act as the leading shock along with rapid development in China. As we can see from the above analysis, these results are in line with those of some previous works, and the mechanism needs to be discussed more deeply in the future.

### 4.3 Asymmetric analysis

The above analysis is based on the assumption of a linear effect of the world oil price on China’s economic growth. However, a number of previous studies found the possibility of an asymmetric impact. Referring to Schorderet [50], we decompose international oil prices into positive increments and negative increments and join the series to the previously constructed vector autoregressive model to consider the nonlinear, asymmetrical effects of international oil prices. In preliminary analysis, we test the stationarity of the transformed series by an augmented Dickey- Fuller (ADF) test. The results show that the decomposed series are stationary. Thus, we can construct the VAR model directly.

The results of the Granger causality test of the decomposed oil price series are reported in Table 5. The results demonstrate that the negative oil price shocks Granger cause China’s industrial economic growth, while positive oil price shocks show no significance. The results appear consistent with the linear one, which also shows the robustness of the test.

**Table 5:** Granger causality test of asymmetry.

Dependent variable	os^+^	os^−^	GEPU	IVA	ALL
os^+^	…	0.044	1.497	0.080	1.786
os^−^	0.079	…	0.622	0.081	1.347
GEPU	0.002	0.217	…	4.950 ^b^	5.086
IVA	0.000	8.811 ^a^	19.104 ^a^	…	33.794 ^a^

Note: The null hypothesis that the variables in a row are not significant in explaining the variables in the column is tested. “ALL” denotes the joint test of the variables.

^a^ Significance at 1%.

^b^ Significance at 5%.

According to the SC information criterion, we choose a one-order lag. The regression results are shown in Table 6. As we can see from the table, the coefficient of negative oil price increase is 1.0283 at a significance level of 99%, which is much larger than the coefficient of positive oil price increase of 0.0106. These results indicate that the impact of oil price decline on China's industrial economic growth has a deeper impact.

**Table 6:** Contemporaneous coefficients in the VAR model.

…	os^+^	os^−^	GEPU	IAV
os^+^ (-1)	-0.0132	-0.0248	-0.0125	0.0106
	(0.0776)	(0.0888)	(0.3015)	(0.4083)
	[-0.1703]	[-0.2803]	[-0.0417]	[0.0259]
os^−^ (-1)	0.0137	0.2736 ^a^	0.1190	1.0283 ^a^
	(0.0658)	(0.0753)	(0.2558)	(0.3464)
	[0.2089]	[3.6328]	[0.4655]	[2.9682]
GEPU (-1)	-0.0129	-0.0095	0.8139 ^a^	-0.2429 ^a^
	(0.0105)	(0.0120)	(0.0410)	(0.0555)
	[-1.2234]	[-0.7889]	[19.8276]	[-4.3708]
IAV(-1)	-0.0028	0.0032	-0.0864 ^b^	0.5854 ^a^
	(0.0100)	(0.0114)	(0.0388)	(0.0526)
	[-0.2821]	[0.2850]	[-2.2247]	[11.1226]
C	0.1064	0.0119	1.0848 ^a^	2.1670 ^a^
	(0.0657)	(0.0751)	(0.2553)	(0.3457)
	[1.6181]	[0.1590]	[4.2487]	[6.2672]

Note: Numbers are the parameters of the matrix *Θ*_*p*_ (see Eq (1)); values in round and square brackets are the p-values and the t-values, respectively.

^a^ Significance at 1%.

^b^ Significance at 5%.

The regression analysis describes the impact of a unit shock of a variable in the system on other variables. Variance analysis is a function that calculates the relative importance of each variable’s shock in the model according to the contribution of each variable in the system, which is decomposed from the predicted mean square error of any endogenous variable. To understand the direction and extent of the positive and negative impacts of oil prices on economic growth deeply, this paper will further analyze the asymmetric impact of oil prices on China's industrial economic growth through variance decomposition.

Table 7 shows the results of the variance decomposition of economic growth. It can be seen from Table 7 that industrial economic growth is most affected by itself. The contribution rate in the 10th period is 69.66%, followed by the uncertainty of global economic policy reaching 22.92% in the 10th period. Regardless of the contribution rate of these two variables, the initial impact of the positive oil price shock on industrial economic growth is greater than the negative impact, at 0.0798% and 0.0007%, respectively. However, the contribution of the negative impact of the oil price in the later period is significantly increased, which is approximately 4.95%, while the positive impact is only 2.47%. The impact of global economic policy uncertainty on industrial economic growth also increased as time passed, reaching 10.92% in the 10th period. This finding shows that global economic policy uncertainty and oil price shock will have an impact on industrial economic growth; the negative impact of oil price decline is greater than the positive impact of oil price increase.

**Table 7:** Variance decomposition of IAV.

Period	S.E.	os^+^	os^−^	GEPU	IAV
1	0.2629	0.0798	0.0007	0.2346	99.6847
2	0.3165	1.4491	3.1158	3.0986	92.3364
3	0.3435	2.0915	4.6372	7.0299	86.2412
4	0.3604	2.3499	5.1275	10.9159	81.6065
5	0.3723	2.4456	5.2208	14.2608	78.0726
6	0.3813	2.4767	5.1867	16.9577	75.3787
7	0.3881	2.4832	5.1212	19.0677	73.3277
8	0.3934	2.4808	5.0560	20.6982	71.7650
9	0.3975	2.4758	4.9998	21.9532	70.5710
10	0.4008	2.4706	4.9541	22.9197	69.6555

Note: Numbers denote percentage of variation in IAV, which can be explained by different oil price shocks and global economic policy uncertainty.

An interesting question is why a decrease of the world oil price is likely to decelerate China’s industrial economic growth, while the influence of an unanticipated increase of the oil price is not significant. In fact, these results can be compared to the findings obtained by Barsky and Kilian [2], Hamilton [57], and Du et al. [34] regarding the increase in oil price being caused by not only the oil demand and supply but also OPEC monopoly pricing, increasing rent and war, etc. When the positive shocks are driven by the economic expansions of US and EU countries, it may not influence China’s exports significantly. Thus, the industrial economic growth is also unaffected. In regard to negative oil shocks, they are more likely to be influenced by the depression of the US and EU countries’ economic activities. In this consideration, a decrease in the oil price may stimulate China’s exports and in turn pull up industrial economic growth. As we can see from the above analysis, the results are in line with those of some previous works, and much work remains for the future.

## 5. Robust analysis

**Fig 3:**
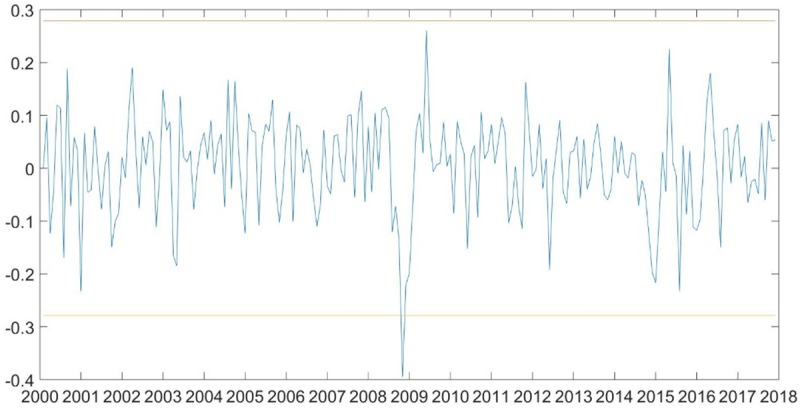
ICSS test of monthly oil returns. Note: Significance at 5%, change points estimated using modified ICSS algorithm.

When the model crossing a long period of time, we should pay close attention to the Structural stability. Considering the breakpoint is unknown, referring to Ewing and Malik [58], we conduct the iterated cumulative sum of squares (ICSS) developed by Inclan and Tiao [59]. This method is algorithm based on IT statistic for testing multiple breaks in the unconditional variance to detect structural breaks in the unconditional variance of oil price returns. The conventional significance is set at 5% level to test for multiple breaks of oil prices. From Fig 3 we can see that there is no breakpoint when oil price series are monthly data, because compared to weekly data and daily data, monthly data drop a lot of information.

To further ensure the reliability of the empirical results, we choose the 2008 financial crisis [15,58,60] as a reference point to examine whether our research is sensitive to the existence of breakpoint. We conduct a dummy variable as an exogenous variable, adding to our VAR model [60]. Table 8 reports the estimate results. We can see from Table 8 that the world oil price shock remains a positive effect on China’s industrial economic growth. It is consistent with our expectation. In addition, the other coefficients in the model have not changed much. Further, Table 9 shows the estimate results of non-linear model (results of non-dummy variable are presented at *4*.*3 asymmetric analysis*), it is in line with our consequence before. Thus, our VAR models are stable and the results are reliable.

**Table 8:** Contemporaneous coefficients in the VAR model.

Dummy variable	No			Yes		
…	R	GEPU	IAV	R	GEPU	IAV
R(-1)	0.1365	0.0659	0.6300 ^a^	0.1360	0.0699	0.6276 ^a^
	(0.0686)	(0.1463)	(0.1811)	(0.0687)	(0.1444)	(0.1808)
	[1.9906]	[0.4506]	[3.4792]	[1.9803]	[0.4839]	[3.4703]
R(-2)	0.0487	-0.1122	0.0284	0.04908	-0.1149	0.0300
	(0.0700)	(0.1491)	(0.1846)	(0.0700)	(0.1472)	(0.1843)
	[0.6965]	[-0.7520]	[0.1538]	[0.7013]	[-0.7807]	[0.1629]
GEPU (-1)	-0.0879 ^a^	0.7814 ^a^	-0.1619	-0.0832 ^a^	0.7481 ^a^	-0.1419
	(0.0326)	(0.0696)	(0.0861)	(0.0332)	(0.0699)	(0.0875)
	[-2.6954]	[11.2271]	[-1.8792]	[-2.5047]	[10.7050]	[-1.6204]
GEPU (-2)	0.0846 ^a^	0.0158	0.0015	0.0887 ^a^	-0.0129	0.0187
	(0.0329)	(0.0703)	(0.0870)	(0.0334)	(0.0702)	(0.0880)
	[2.5687]	[0.2243]	[0.0172]	[2.6544]	[-0.1832]	[0.2125]
IAV(-1)	-0.0063	-0.0214	0.3273 ^a^	-0.0087	-0.0046	0.3172 ^a^
	(0.0238)	(0.0508)	(0.0628)	(0.0240)	(0.0505)	(0.0633)
	[-0.2654]	[-0.4207]	[5.2080]	[-0.3613]	[-0.0913]	[5.0119]
IAV(-2)	0.0249	-0.0922	0.4198 ^a^	0.0224	-0.0747	0.4092 ^a^
	(0.0230)	(0.0492)	(0.0608)	(0.0233)	(0.0490)	(0.0614)
	[1.0788]	[-1.8751]	[6.8995]	[0.9611]	[-1.5241]	[6.6693]
C	-0.0258	1.2252 ^a^	1.3527 ^a^	-0.0610	1.4749 ^a^	1.2026 ^a^
	(0.1344)	(0.2868)	(0.3549)	(0.1423)	(0.2992)	(0.3748)
	[-0.1917]	[4.2725]	[3.8117]	[-0.4288]	[4.9301]	[3.2089]
C1	…	…	…	0.0132	-0.0936 ^a^	0.0562
	…	…	…	(0.0173)	(0.0364)	(0.0456)
	…	…	…	[0.7620]	[-2.5692]	[1.2325]

Note: Numbers are the parameters of the matrix *Θ*_*p*_ (see Eq (1)); values in round and square brackets are the p-values and the t-values, respectively.

^a^ Significance at 1%.

**Table 9:** Contemporaneous coefficients in the VAR model (asymmetric).

…	os^+^	os^−^	GEPU	IAV
os^+^ (-1)	-0.0304	-0.0266	0.1085	-0.0843
	(0.0776)	(0.0889)	(0.2973)	(0.4038)
	[-0.3917]	[-0.2990]	[0.3650]	[-0.2087]
os^−^ (-1)	0.0269	0.2746 ^a^	0.0245	1.1062 ^a^
	(0.0659)	(0.0755)	(0.2525)	(0.3430)
	[0.4084]	[3.6367]	[0.0972]	[3.2246]
GEPU (-1)	-0.0060	-0.0110	0.7542 ^a^	-0.1740 ^a^
	(0.0121)	(0.0138)	(0.0463)	(0.0629)
	[-0.4927]	[-0.7973]	[16.2936]	[-2.7667]
IAV(-1)	-0.0081	0.0033	-0.0461	0.5478 ^a^
	(0.0104)	(0.0119)	(0.0400)	(0.0542)
	[-0.7818]	[0.2793]	[-1.1546]	[10.1012]
C	0.0819	0.0197	1.3060 ^a^	1.8911 ^a^
	(0.0694)	(0.0796)	(0.2661)	(0.3614)
	[1.1807]	[0.2472]	[4.9088]	[5.2325]
C1	0.0128	-0.0015	-0.1038 ^a^	0.1092
	(0.0093)	(0.0107)	(0.0358)	(0.0486)
	[1.3762]	[-0.1386]	[-2.9025]	[2.2470]

Note: Numbers are the parameters of the matrix *Θ*_*p*_ (see Eq (1)); values in round and square brackets are the p-values and the t-values, respectively.

^a^ Significance at 1%.

**Fig 4:**
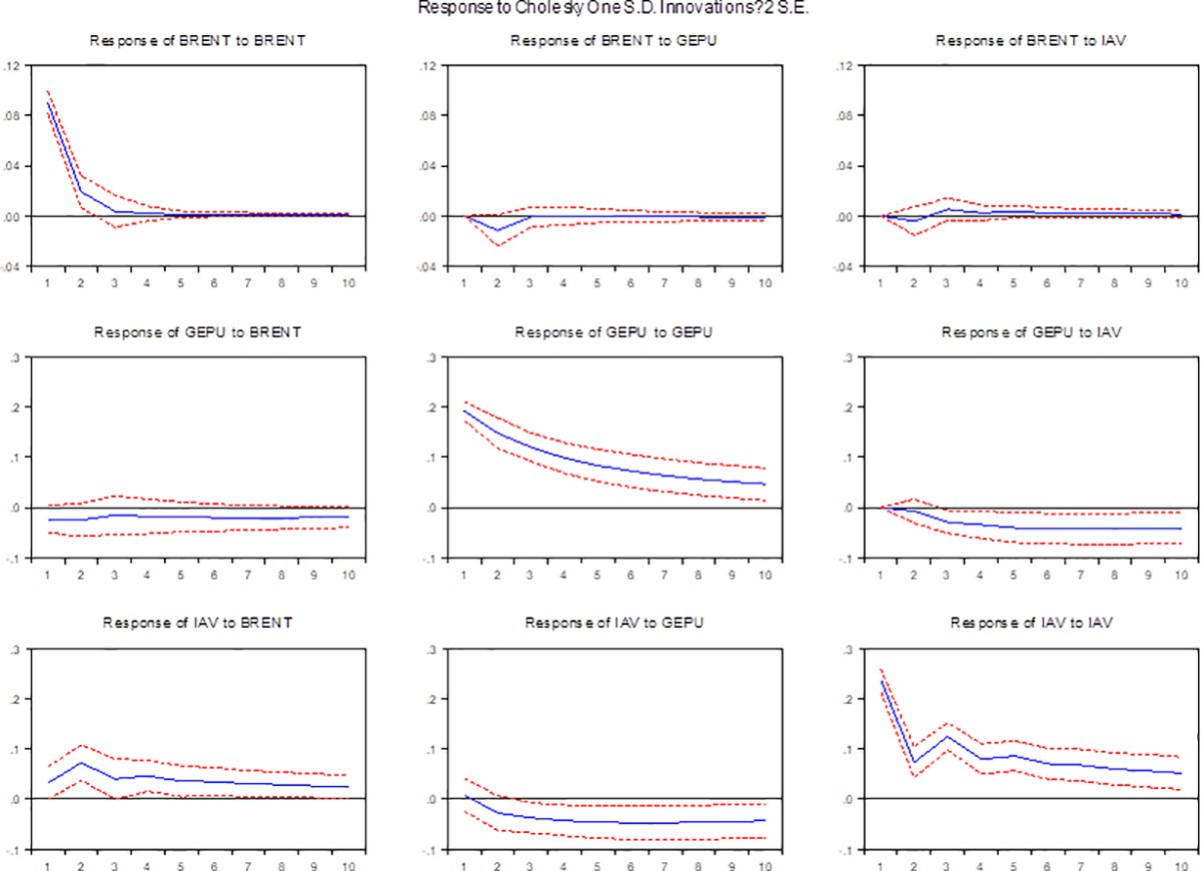
Impulse response functions of BRENT. Note: In VAR (R, GEPU, IAV), GEPU and IAV are the global economic policy uncertainty and China’s industrial added value in first log difference, and R is real stock return of world real oil price (BRENT). The horizontal axis is the period. The vertical axis is the explanation level of the dependent variables to independent variables. In the model, we fix the periods at 10 months.

**Fig 5:**
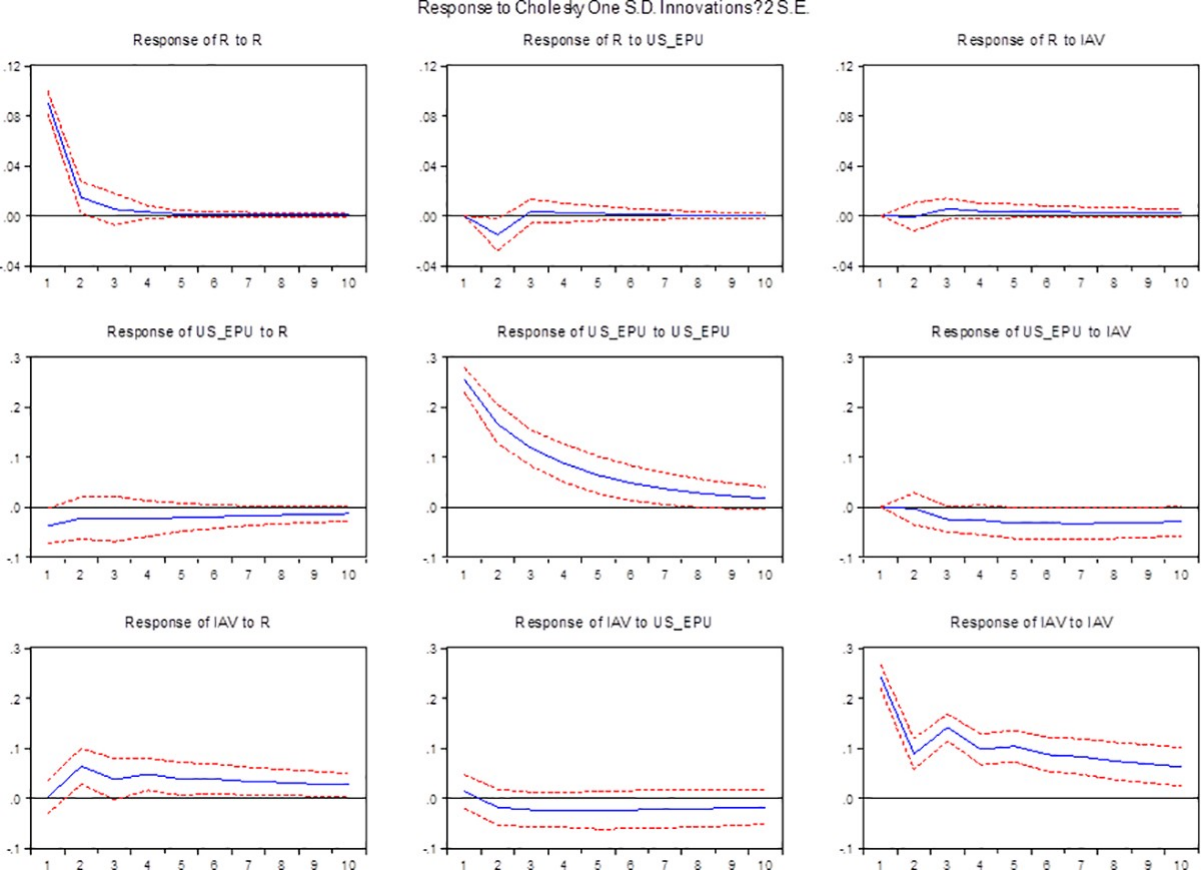
Impulse response functions of US-EPU. Note: In VAR (R, US-EPU, IAV), US-EPU and IAV are the global economic policy uncertainty and China’s industrial added value in first log difference, and R is real stock return of world real oil price (WTI). The horizontal axis is the period. The vertical axis is the explanation level of the dependent variables to independent variables. In the model, we fix the periods at 10 months.

The robustness is always an issue when we examine the effectiveness of the results, especially for those that may be influenced by the variable selections. To obtain a robust evaluation of the model results, we conduct robustness checks. First, we choose two alternative variables, (i) an alternative oil price and (ii) an alternative measure of economic policy uncertainty, and construct VAR models in the next section. We made the same treatment on alternative variables—first log difference of monthly stock price (BRENT) and log transformation of economic policy uncertainty. Tables A and B in S1 File shows the statistical description and unit root test of two alternative variables. The results of the primary test show that both two variables do not have a unit root at a 1% level of significance. Thus, we accept the assumption that in log levels, the variables are I (0) processes. Figs 4 and 5 report the impulse results. To save place, we only present the impulse responses results of robust test. The results of other steps will be shown in the appendix.

The Brent Crude price, a major trading classification of sweet light crude oil that accounts for more than two-thirds of the world's crude oil, serves as a major benchmark price for purchases of oil worldwide, holding a spread and lead-lag relationship with WTI. In this situation, the exchange of world oil price should not make any difference. From Table C in S1 File, except for the difference in Granger causality between GEPU and R, the Granger causality among other variables remains unchanged. Fig 4 presents the impulse response function of Brent Crude. Obviously, no differences are found in the impulse response analysis. The results are consistent with the original results, showing strong support for our analysis.

As Wei et al. [61] described in their work, despite reflecting the foundation of the supply and demand relationship, the EPU index also impacts the expectations of participants and increases their speculation. Therefore, the global economic policy uncertainty index contains more information, and its prediction accuracy of oil prices is more remarkable. Considering the important position of the United States in the world, the EPU index of the US is superior to those of other countries and contains more information than the global EPU index. Thus, we choose US-EPU to perform the robustness test. Table D in S1 File presents the Granger causality test of US-EPU. The Granger causality become weaken when economic policy uncertainty is alternated. It may because since China joined the World Trade Organization in 2001, the multilateral trade systems make US become not so important. But US still carry a big weight in the world. Fig 5 depicts the impulse responses applying US-EPU. Similarly, the results are stable after running the same VAR model with the US-EPU.

Further, we examine the non-linear relationships with the oil price alternated by BRENT, Tables E and G in S1 File display the results of Granger causality test, Contemporaneous coefficients and Variance decomposition respectively. These results are consistent with the previous, which means that the non-linear model is robust.

## 6. Conclusion

Different from the previous studies of the impact of oil price shocks on China's economic growth, this paper systematically integrates the world oil price, global economic policy uncertainty and economic growth. Using monthly data on oil prices and economic growth, we construct a VAR model, empirically analyze the impact of international oil prices and economic policy uncertainty on China's industrial economic growth, and reach the following main conclusions.

The Granger causality test show that the oil price and the economic policy uncertainty significantly granger cause China’s industrial economic growth. The impulse-response function shows that there is a mutually inhibiting relationship between international oil prices and economic policy uncertainty, both of them response negatively to each other’s shock. Moreover, the increase of economic policy uncertainty will suppress China’s industrial economic policy uncertainty while oil price shocks promote. Results of non-linear model show that there is a significant asymmetry in oil prices shocks. The drops in oil prices have a greater influence on China’s industrial economic growth while the positive shocks are not significant. These results are robust and stability. Through the above analysis, this paper argues that oil prices, economic uncertainty and China's economic growth are not isolated, but closely related and mutually influential.

These conclusions have three important implication to policy-makers. First, policy-makers need to pay more attention to economic when oil price goes down. Second, to maintain a stable environment for economic growth, policy-makers should keep eyes on the world, and improve resilience to uncertain events. Third, in order to ensure the healthy development of the domestic oil market, we should complete the domestic oil pricing mechanisms such as bring global economic policy uncertainty into the consideration.

## Supporting information

S1 File (DOCX)
