# Supplementary material for: Oil price shocks, economic policy uncertainty and industrial economic growth in China
Source: PLoS One. 2019 May 10;14(5):e0215397. doi: 10.1371/journal.pone.0215397 (PMC6510409; doi:10.1371/journal.pone.0215397)
Supplement: S1 File — (DOCX) [file pone.0215397.s001.docx]

**Table A. Statistical description**

| Variables | Mean | Median | (Max, min) | Std. Dev. | Skewness | Kurtosis |
| --- | --- | --- | --- | --- | --- | --- |
| BRENT | 0.0043 | 0.0191 | (0.1979, -0.3110) | 0.0921 | -0.8973 | 4.2021 |
| US-GEPU | 4.7249 | 4.6981 | (5.6478,3.8018) | 0.3703 | 0.0551 | 2.7082 |
| $b\mathrm{os}^{+}$ | 0.0373 | 0.0191 | (0.1979,0) | 0.0460 | 1.2603 | 4.0847 |
| ${b\mathrm{os}}^{-}$ | -0.0330 | 0 | (0, -0.3110) | 0.0624 | -2.3996 | 8.6627 |

Note: All variables are expressed as natural log, expect for BRENT.

**Table B. Unit root test**

| Variables | ADF | | | | PP | | | |
| --- | --- | --- | --- | --- | --- | --- | --- | --- |
|  | 5% Level | 1% Level | | t-Statistic | 5% Level | 1% Level | | t-Statistic |
| BRENT | -2.8749 | -3.4609 | -11.7719 ^a^ | | -2.8749 | -3.4609 | -11.7669 ^a^ | |
| US-EPU | -2.8748 | -3.4607 | -5.8679 ^a^ | | -2.8748 | -3.4607 | -5.6355 ^a^ | |

Note: The null hypothesis of the ADF test/PP test is that the series has a unit root.

^a^ Signiﬁcance at 1%.

**Table C. Granger causality test (BRENT)**

| Dependent variable | BRENT | GEPU | IVA | ALL |
| --- | --- | --- | --- | --- |
| BRENT | … | 3.412 | 1.594 | 5.759 |
| GEPU | 0.740 | … | 8.094 ^b^ | 8.366 ^c^ |
| IVA | 12.206 ^a^ | 10.023 ^a^ | … | 24.829 ^a^ |

Note: The null hypothesis that the variables in a row are not significant in explaining the variables in the column is tested. “ALL” denotes the joint test of the variables.

^c^ Signiﬁcance at 10%.

^b^ Signiﬁcance at 5%.

^a^ Signiﬁcance at 1%

**Table D. Granger causality test (US-EPU)**

| Dependent variable | R | US-EPU | IVA | ALL |
| --- | --- | --- | --- | --- |
| R | … | 6.802 ^c^ | 1.781 | 8.786 |
| US-EPU | 0.150 | … | 4.481 | 4.818 |
| IVA | 12.206 ^a^ | 3.137 | … | 18.445 ^a^ |

Note: The null hypothesis that the variables in a row are not significant in explaining the variables in the column is tested. “ALL” denotes the joint test of the variables.

^c^ Signiﬁcance at 10%.

^a^ Signiﬁcance at 1%

**Table E. Granger causality test of asymmetry**

| Dependent variable | $b\mathrm{os}^{+}$ | b$\mathrm{os}^{-}$ | GEPU | IVA | ALL |
| --- | --- | --- | --- | --- | --- |
| $b\mathrm{os}^{+}$ | … | 0.899 | 4.754 ^b^ | 1.721 | 5.659 |
| $b\mathrm{os}^{-}$ | 0.001 | … | 0.105 | 0.184 | 0.601 |
| GEPU | 0.590 | 0.385 | … | 4.971 ^b^ | 5.543 |
| IVA | 1.613 | 12.083^***^ | 21.368 ^a^ | … | 34.866 ^a^ |

Note: The null hypothesis that the variables in a row are not significant in explaining the variables in the column is tested. “ALL” denotes the joint test of the variables.

^b^ Signiﬁcance at 5%.

^a^ Signiﬁcance at 1%.

**Table F. Contemporaneous coefficients in the VAR model (BRENT)**

| … | $b\mathrm{os}^{+}$ | $\mathrm{bos}^{-}$ | GEPU | IAV |
| --- | --- | --- | --- | --- |
| $b\mathrm{os}^{+}$ (-1) | 0.0662 | 0.0031 | -0.2461 | -0.5502 |
|  | (0.0756) | (0.0975) | (0.3203) | (0.4332) |
|  | [ 0.8748] | [ 0.0323] | [-0.7685] | [-1.2700] |
| $\mathrm{bos}^{-}$ (-1) | -0.0536 | 0.3368 ^a^ | 0.1484 | 1.1249^***^ |
|  | (0.0565) | (0.0728) | (0.2392) | (0.3236) |
|  | [-0.9482] | [ 4.6250] | [ 0.6202] | [ 3.4760] |
| GEPU (-1) | -0.0210 | -0.0040 | 0.8108 ^a^ | -0.2553 ^a^ |
|  | (0.0096) | (0.0124) | (0.0408) | (0.0552) |
|  | [-2.1804] | [-0.3247] | [ 19.8596] | [-4.6225] |
| IAV(-1) | -0.0121 | 0.0051 | -0.0871 | 0.5727 ^a^ |
|  | (0.0092) | (0.0119) | (0.0391) | (0.0529) |
|  | [-1.3119] | [ 0.4290] | [-2.2297] | [ 10.8362] |
| C | 0.1600 ^a^ | -0.0157 | 1.1108 ^a^ | 2.2772 ^a^ |
|  | (0.0600) | (0.0774) | (0.2542) | (0.3439) |
|  | [2.6656] | [ -2.025] | [4.3695] | [6.6225] |

Note: Numbers are the parameters of the matrix $\Theta_{p}$ (see Eq. (1)); values in round and square brackets are the p-values and the t-values, respectively.

^a^ Signiﬁcance at 1%.

**Table G. Variance decomposition of IAV (BRENT)**

| Period | S.E. | b$\mathrm{os}^{+}$ | b$\mathrm{os}^{-}$ | GEPU | IAV |
| --- | --- | --- | --- | --- | --- |
| 1 | 0.2623 | 2.9508 | 0.0102 | 0.1630 | 96.8760 |
| 2 | 0.3152 | 3.3260 | 4.4243 | 3.1616 | 89.0882 |
| 3 | 0.3439 | 3.9200 | 6.9929 | 6.8285 | 82.2585 |
| 4 | 0.3618 | 4.2398 | 8.0023 | 10.336 | 77.4214 |
| 5 | 0.3740 | 4.3733 | 8.3101 | 13.3519 | 73.9647 |
| 6 | 0.3829 | 4.4175 | 8.3509 | 15.8022 | 71.4294 |
| 7 | 0.3896 | 4.4242 | 8.3025 | 17.7360 | 69.5374 |
| 8 | 0.3947 | 4.4167 | 8.2325 | 19.2406 | 68.1103 |
| 9 | 0.3986 | 4.4052 | 8.1646 | 20.4043 | 67.0259 |
| 10 | 0.4016 | 4.3937 | 8.1062 | 21.3028 | 66.1973 |

Note: Numbers denote percentage of variation in IAV, which can be explained by different oil price shocks and global economic policy uncertainty.
